# Enteroaggregative *Escherichia coli* Adherence Fimbriae Drive Inflammatory Cell Recruitment via Interactions with Epithelial MUC1

**DOI:** 10.1128/mBio.00717-17

**Published:** 2017-06-06

**Authors:** Erik J. Boll, Jorge Ayala-Lujan, Rose L. Szabady, Christopher Louissaint, Rachel Z. Smith, Karen A. Krogfelt, James P. Nataro, Fernando Ruiz-Perez, Beth A. McCormick

**Affiliations:** a Department of Microbiology and Physiological Systems, University of Massachusetts Medical School, Worcester, Massachusetts, USA; b Department of Pediatrics, University of Virginia School of Medicine, Charlottesville, Virginia, USA; c Department of Microbiology and Infection Control, Statens Serum Institut, Copenhagen, Denmark; Pasteur Institute

**Keywords:** *Escherichia coli*, fimbriae, inflammation, mucin

## Abstract

Enteroaggregative *Escherichia coli* (EAEC) causes diarrhea and intestinal inflammation worldwide. EAEC strains are characterized by the presence of aggregative adherence fimbriae (AAF), which play a key role in pathogenesis by mediating attachment to the intestinal mucosa and by triggering host inflammatory responses. Here, we identify the epithelial transmembrane mucin MUC1 as an intestinal host cell receptor for EAEC, demonstrating that AAF-mediated interactions between EAEC and MUC1 facilitate enhanced bacterial adhesion. We further demonstrate that EAEC infection also causes elevated expression of MUC1 in inflamed human intestinal tissues. Moreover, we find that MUC1 facilitates AAF-dependent migration of neutrophils across the epithelium in response to EAEC infection. Thus, we show for the first time a proinflammatory role for MUC1 in the host response to an intestinal pathogen.

## IMPORTANCE

EAEC is a clinically important intestinal pathogen that triggers intestinal inflammation and diarrheal illness via mechanisms that are not yet fully understood. Our findings provide new insight into how EAEC triggers host inflammation and underscores the pivotal role of AAFs—the principal adhesins of EAEC—in driving EAEC-associated disease. Most importantly, our findings add a new dimension to the signaling properties of the transmembrane mucin MUC1. Mostly studied for its role in various forms of cancer, MUC1 is widely regarded as playing an anti-inflammatory role in response to infection with bacterial pathogens in various tissues. However, the role of MUC1 during intestinal infections has not been previously explored, and our results describe the first report of MUC1 as a proinflammatory factor following intestinal infection.

## INTRODUCTION

Enteroaggregative *Escherichia coli* (EAEC) causes endemic and sporadic diarrheal illness throughout the world (1–4). Several EAEC-associated disease outbreaks have been reported (5–7), including a large food-borne outbreak caused by a Shiga toxin (Stx)-producing EAEC strain in May 2011 in Germany leading to 3,842 confirmed cases and 54 deaths (8, 9). Asymptomatic colonization by EAEC has been linked to subclinical inflammation and growth retardation (10).

Orally ingested EAEC bacteria adhere to the intestinal mucosa and form biofilms, thus allowing the bacteria to persistently colonize the intestinal tract (11). The specific factors contributing to EAEC interaction with the intestinal mucosa are not fully understood. However, aggregative adherence fimbriae (AAF), the principal adhesins of EAEC, are thought to play a crucial role. Their involvement in biofilm formation, adherence to intestinal epithelial cells, and triggering of host inflammatory responses has been well studied (12–15).

Five variants of the AAF major structural subunits have been described thus far: AggA (AAF/I), AafA (AAF/II), Agg3A (AAF/III), Agg4A (AAF/IV), and Agg5A (16–19). The AAFs are encoded on the large pAA virulence plasmid along with AggR, a transcription factor regulating AAF biogenesis, and other putative EAEC virulence factors (11). The AAFs belong to the Afa/Dr family of adhesins, which assemble via the usher/chaperone pathway (20). The AAF structure comprises a major subunit (A) and a putative minor subunit (B). The major subunits of different AAF variants share only ca. 25% amino acid identity, but they are all positively charged, thus distinguishing them from other members of the Afa/Dr family. This feature is thought to play a role in EAEC adhesion (21).

EAEC does not invade the epithelial barrier, and so direct interactions between EAEC and the intestinal epithelium are likely restricted to the apical surfaces of the cells. An apically located cell receptor for EAEC has yet to be identified, although potential binding partners have been found. The major pilin subunits of AAFs have been shown to bind to extracellular matrix proteins, such as the glycoproteins fibronectin and laminin, as well as cytokeratin 8 (22, 23). However, given that none of these compounds are present at the apical surface of healthy epithelium, other as yet unidentified receptors likely mediate initial bacterial contact.

In the healthy human intestine, the epithelial cells are separated from the commensal microbiota by a thick mucous barrier, which serves as a first line of innate defense. In the colon, the expanded outer mucus layer is rich in secreted mucins, which are large glycoproteins that assemble into oligomeric structures. Mucins are heavily *O*-glycosylated, decorated with sialic acid, galactose, fucose, *N*-acetylglucosamine (GlcNAc), and *N*-acetylgalactosamine (GalNAc) glycans that vary in length and degree of branching. These complex oligosaccharides serve as attachment sites and as a potential source of nutrients for the microbiota (24). While the outer mucus layer entraps bacteria, the inner mucus layer is thinner, and typically, bacteria do not penetrate this layer (25). Transmembrane mucins are a key component of the inner mucus layer and consist of a large extracellular *O*-glycosylated domain linked noncovalently to a cell membrane-spanning domain, with a cytoplasmic tail that tethers the mucins to the epithelial surface (26). Transmembrane mucins reach further out into the intestinal lumen than any other membrane protein and are likely to be the first point of direct contact between host cells and pathogens capable of penetrating the secreted mucus layer, such as EAEC (27).

Like secreted mucins, transmembrane mucins may act as releasable decoy molecules on the apical surface to limit bacterial adherence, infection, and inflammation (28, 29). The oligosaccharides are attached to tandem repeats of amino acids within the mucin backbone and may also serve as ligands for microbial adhesins (30, 31). Additionally, transmembrane mucins serve as sensors and regulators of the external milieu and transmit signals through posttranslational modifications of their cytoplasmic tails (32, 33). Deregulated transmembrane mucin production and signaling have been associated with numerous types of cancers and inflammatory disorders (34).

MUC1 is a transmembrane mucin expressed by all human glandular epithelial tissues and throughout the gastrointestinal tract at low levels (35). This mucin received extensive attention in cancer research due to its aberrant expression at high levels in human carcinomas and in inflamed tissue (26, 28, 36–38). Furthermore, MUC1 has also been shown to act as a cell receptor for various pathogenic organisms, including *Pseudomonas aeruginosa*, *Campylobacter jejuni*, and *Helicobacter pylori* (28, 39, 40).

In this study, we show that transmembrane mucins are receptors for the AAF adhesins of EAEC on the intestinal epithelium. Moreover, we demonstrate that the AAFs elicit intestinal inflammation through MUC1-mediated host cell signaling.

## RESULTS

### MUC1 is a cell receptor for EAEC.

Previous studies have shown that EAEC strains evoke host inflammation through AAF-mediated binding to the intestinal epithelial surface (12, 13). We have shown that EAEC strain 042 induces transepithelial migration of polymorphonuclear neutrophils (PMNs) across polarized T84 cell monolayers by activating apical release of an eicosanoid-based PMN chemoattractant, which guides PMNs across the intestinal epithelium (41). EAEC strains lacking AAF were unable to trigger host cell inflammatory signaling, suggesting that this process is likely mediated by binding of AAF to an unknown apical surface receptor (13).

We have previously reported that AAF adhesins induce hemagglutination; the species-specific hemagglutination pattern differs among the fimbrial variants (17). We have shown that AAF/II causes temperature-dependent hemagglutination of human type A erythrocytes (42). Since some diarrheagenic *E. coli* adhesins recognize sialylated receptors, we tested whether hemagglutination in AAF/II-producing strain 042 would be reduced by treatment of the erythrocytes with neuraminidase. Treatment of erythrocytes with neuraminidase for 20 min completely abolished the hemagglutination phenotype (not shown).

**FIG 1:**
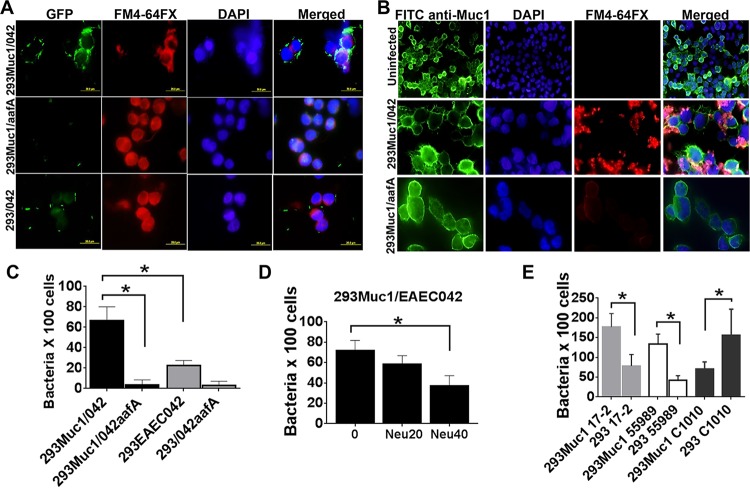
MUC1 is a cell receptor for EAEC. (A) HEK293 cells transfected with pcDNA3 or pcDNA3-MUC1 were infected in suspension with EAEC strain 042 or with its isogenic fimbrial mutant (042*aafA*), each expressing a GFP reporter and analyzed by fluorescence microscopy (FM). Cell membranes were stained with FM4-64FX, and DNA was stained with DAPI. (B) Uninfected and EAEC 042-infected HEK293pMUC1 cells were stained with FITC-conjugated anti-MUC1 MAb and DAPI and analyzed by FM. Bacteria were stained with M4-FX64. (C) HEK293-pcDNA-MUC1 cells were infected in suspension as described above in panel A, and bacteria that adhered to cells were enumerated microscopically. (D) HEK293-pcDNA3-MUC1 cells were treated with two concentrations (20 U and 40 U) of neuraminidase (Neu) for 1 h before infection with strain 042 followed by quantification of bacterial adherence as described above for panel C. (E) HEK293 cells not expressing or expressing recombinant MUC1 were infected with EAEC strain 17-2 (which expresses AAF/I), 55989 (AAF/III), or C1010-00 (AAF/VI), followed by quantification of bacterial adherence as described above for panel C. Bars represent the means of at least three independent experiments, with the error bars indicating standard deviations (SD) (error bars). Values that are significantly different (*P* < 0.05) are indicated by a bar and asterisk.

As our data suggested that AAF/II binds to a sialylated receptor on erythrocytes, we hypothesized that the AAF/II receptor on epithelial cells would be a sialylated glycoprotein; the most abundant membrane-associated glycoproteins on human epithelial cell surfaces are the transmembrane mucins. To further investigate the potential involvement of mucins in mediating EAEC binding and/or inflammation, we focused our attention on the transmembrane mucin MUC1, which acts as an adhesion target for pathogens, including *Pseudomonas aeruginosa*, *Campylobacter jejuni*, and *Helicobacter pylori* (28, 29, 39). To determine whether MUC1 could also serve as a receptor for EAEC, we evaluated bacterial adhesion to healthy HEK293 cells that do not express MUC1 compared to HEK293 cells stably expressing human MUC1 (see Fig. S1 in the supplemental material). Both the wild-type EAEC 042 (wt 042) strain and the 042Δ*aafA* strain expressed a green fluorescent protein (GFP) reporter, and bacterial adhesion was quantified by fluorescence microscopy. We found that the 042 wt strain bound at significantly higher levels to MUC1-positive HEK293 cells than to MUC1-negative cells, whereas the 042Δ*aafA* strain failed to bind to either cell line (Fig. 1A and C). Moreover, as shown in Fig. 1B, we observed colocalization of 042 wt strain with MUC1 on the surfaces of MUC1-positive HEK293 cells.

10.1128/mBio.00717-17.1 FIG S1  HEK293pcDNA3 and HEK293pcDNA3-MUC1 cells were stained with FITC-conjugated anti-MUC1 Mab and DAPI and analyzed by fluorescence microscopy (FM). *MUC1* gene expression in these cells was analyzed by quantitative reverse transcription-PCR (qRT-PCR). Download FIG S1, PDF file, 0.7 MB. Copyright © 2017 Boll et al. 2017 Boll et al. This content is distributed under the terms of the Creative Commons Attribution 4.0 International license.

MUC1 oligosaccharides carry terminal sialic acids (35). To determine the importance of sialic acid residues on MUC1 for binding of EAEC, we pretreated MUC1-positive HEK293 cells with neuraminidase for 1 h prior to infection with the 042 wt strain. As shown in Fig. 1D, this treatment significantly reduced bacterial binding, suggesting that sialic acid is required for recognition of MUC1 by AAF.

EAEC strain 042 expresses AAF variant II, one of five subtypes that share a low level of similarity. We therefore examined whether the interaction with MUC1 was specific to this fimbrial subtype. HEK293-MUC1 cells were infected with EAEC strains expressing other AAF variants, including strains 17-2 (AAF/I), 55989 (AAF/III), and C1010-00 (AAF/IV). The presence of MUC1 significantly enhanced binding of strains 17-2 and 55989 to HEK293 cells (Fig. 1E). Surprisingly, the opposite effect was observed with strain C1010-00.

Collectively, these findings demonstrate that MUC1 is a host cell receptor for EAEC strains and that binding is mediated by specific AAFs. This binding is at least partially dependent on MUC1 sialic acid residues.

### EAEC binds to MUC1 on the surfaces of intestinal epithelial cells.

We next investigated whether these findings in a transfected cell line extended to bacterial binding of native MUC1 expressed on the epithelial surface. Human colon cancer-derived T84 cells produce mucins (including MUC1), polarize to form tight epithelial barriers, and serve as a useful model of intestinal epithelia. EAEC bacteria adhere to the apical surfaces of polarized T84 cells and can induce neutrophil transmigration in the physiologically relevant basolateral-to-apical direction (41). We were able to knock down MUC1 expression in T84 cells using lentiviral short hairpin RNA (shRNA) constructs targeting MUC1. Of the five constructed *MUC1* shRNA cell lines, we chose one with a significant decrease in MUC1 expression at both the mRNA and protein levels (Fig. S2) for further experimentation.

10.1128/mBio.00717-17.2 FIG S2  (A and B) Whole-cell lysates of scrambled control T84 cells or cells with MUC1 shRNA (A) or MUC13 shRNA (B) were separated on gradient polyacrylamide gels and immunoblotted using MUC1 (VU4H5) Mab or MUC13 Mab antibody. Actin levels served as a control for protein loading conditions. (C and D) mRNA was extracted from scrambled control T84 cells or cells with MUC1 shRNA (C) or MUC13 shRNA (D) using an RNeasy minikit (Qiagen) and converted to cDNA using a QuantiTect reverse-transcription kit (Qiagen). Transcriptional levels of MUC1 and MUC13 were then quantified by qRT-PCR. (E and F) Fixed scrambled control T84 cells or cells with MUC1 shRNA (E) or MUC13 shRNA (F) grown in Lab-Tek chamber slides (Nunc) were blocked with PBS containing 1% BSA and 0.1% Triton X-100, incubated with MUC1 (VU4H5) Mab (Abcam) (1:100) or MUC13 (Abcam) (1:100) Mab, washed, and incubated with Alexa Fluor 633. After the cells were washed, the cells were mounted using DAPI-containing Vectashield solution (Vector), and analyzed using a fluorescence microscope. Download FIG S2, PDF file, 1.5 MB. Copyright © 2017 Boll et al. 2017 Boll et al. This content is distributed under the terms of the Creative Commons Attribution 4.0 International license.

**FIG 2:**
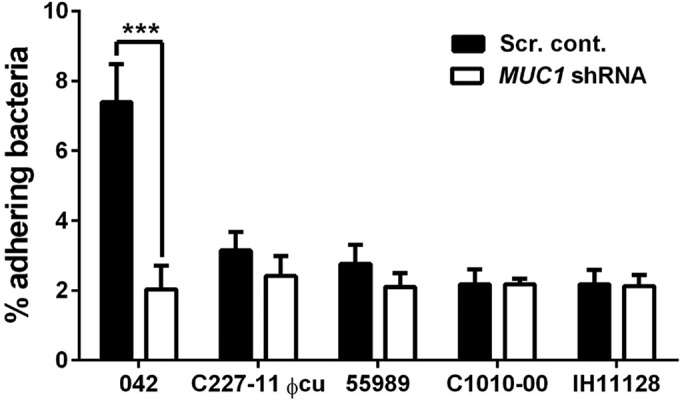
EAEC binds to MUC1 on the surfaces of intestinal epithelial cells. Monolayers of scrambled control (Scr. cont.) T84 cells or cells with MUC1 shRNA were infected with EAEC strain 042, C227-11 ϕcu (AAF/I), 55989, or C1010-00 or diffusely adherent *E*. *coli* (DAEC) strain IH11128. Cell-adhered bacteria were then enumerated by plating. Bars represent the means of at least three independent experiments plus SD. ***, *P* < 0.001.

T84 MUC1 shRNA cells were infected with the different EAEC strains described above as well as the AAF/I-expressing Stx-EAEC German outbreak strain cured of its Stx-encoding phage (C227-11 φcu) (43). For a control, we also included diffusely adhering *E. coli* strain IH11128. IH11128 expresses Dr fimbriae, which are related to AAF fimbriae but bind specifically to decay-accelerating factor (DAF)/CD55 on the surfaces of T84 cells, and therefore should not be affected by the lack of MUC1 (44). The 042 strain adhered significantly less to T84 MUC1 shRNA cells than to control T84 cells (Fig. 2). In contrast, MUC1 knockdown did not significantly affect binding of strain C227-11 ϕcu, 55989, or C1010-00 or the diffusely adherent *E. coli* IH11128 strain.

### EAEC-induced PMN migration is mediated by MUC1.

Transmembrane mucins are increasingly recognized for their involvement in host cell signaling cascades via their cytoplasmic tails (34). MUC1 has been shown to play an anti-inflammatory role in response to lung and gastric infections (33, 40, 45). However, its role in response to intestinal infection has not been determined. Given that AAFs are critical for induction of inflammation by EAEC, we sought to determine whether the interaction between AAFs and MUC1 triggers EAEC-induced inflammation. T84 MUC1 shRNA cells were infected with various EAEC strains, and the ability to induce neutrophil transepithelial migration was measured.

**FIG 3:**
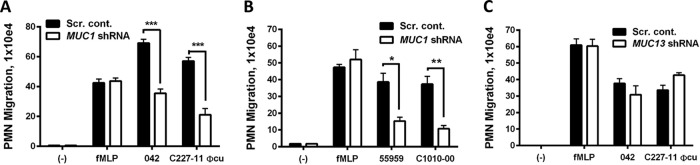
MUC1 is involved in EAEC-induced PMN migration. (A to C) Monolayers of scrambled control T84 cells or cells with MUC1 shRNA (A and B) or MUC13 shRNA (C) were infected apically with EAEC strains. HBSS^+^ buffer (-) and the PMN chemoattractant *N*-formyl-methionyl-leucyl-phenylalanine (fMLP) were included as negative and positive controls, respectively. The data are expressed as the means plus SD for at least triplicate samples, and the data shown are from one representative experiment of at least three independent experiments performed with similar results. ***, *P* < 0.001; **, *P* < 0.01; *, *P* < 0.05.

Reduction of *MUC1* expression profoundly reduced the ability of EAEC strains 042 and C227-11 ϕcu to induce PMN migration compared with infection of control cells (Fig. 3A). When we infected MUC1 knockdown cells with EAEC type strains 55989 and C1010-00, we observed reduced inflammatory migration similar to the phenotypes of 042 and C227-11 ϕcu (Fig. 3B). Collectively, these results indicate that MUC1 is involved in EAEC-induced inflammation regardless of the AAF variant expressed.

In addition to MUC1, several other transmembrane mucins such as MUC3, MUC4, and MUC13 are expressed in the gastrointestinal tract (34). MUC13 is highly expressed in healthy colonic epithelium, where EAEC has been shown to bind abundantly (46, 47). Moreover, MUC13 has been implicated in intestinal inflammation (33). We therefore sought to determine whether MUC13 also acts as an inflammatory receptor for EAEC. A T84 MUC13 shRNA cell line with greatly reduced MUC13 expression was generated (Fig. S2). MUC1 expression was not altered in the T84 MUC13 shRNA cell line and vice versa (data not shown). As shown in Fig. 3C, MUC13 knockdown did not affect the ability of strain 042 or C227-11 ϕcu to trigger neutrophil transepithelial migration, suggesting that the AAFs specifically trigger inflammation through MUC1.

### Purified AAFs elicit MUC1-dependent inflammation in the presence of commensal *E. coli.*

**FIG 4:**
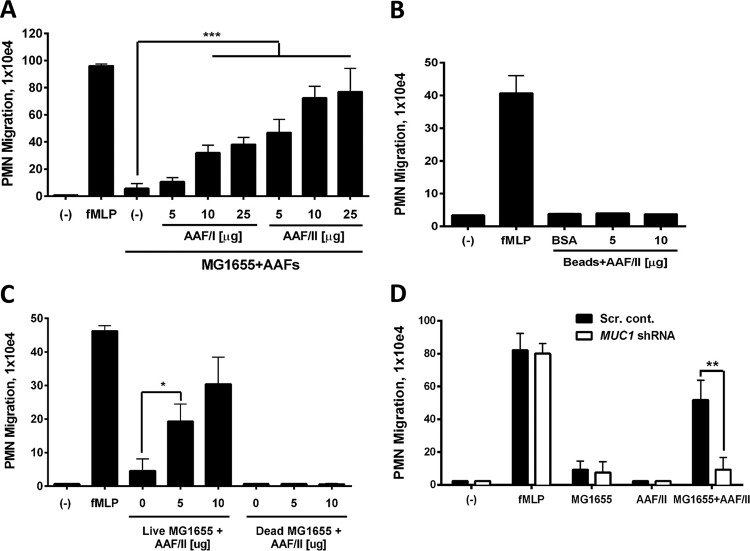
Purified AAFs elicit MUC1-dependent PMN transepithelial migration in the presence of commensal *E. coli*. (A and C) T84 cell monolayers were infected with live or heat shock-killed commensal *E. coli* strain MG1655 in the presence or absence (-) of AAF/I or AAF/II, respectively, and assessed for PMN transmigration. (B) T84 monolayers were exposed to beads coated with purified AAF/II or coated with BSA as a negative control and assessed for PMN migration. (D) Monolayers or scrambled control or MUC1 shRNA T84 cells were infected with live *E*. *coli* MG1655 in the presence of 10 μg AAF/II and assessed for PMN transmigration. The data are expressed as the means plus SD for at least triplicate samples and represent one of at least three independent experiments performed with similar results. ***, *P* < 0.001; **, *P* < 0.01; *, *P* < 0.05.

Having shown that AAFs bind to MUC1 and that MUC1 contributes to neutrophil infiltration, we next sought to establish a direct link between AAFs and MUC1-induced inflammation. To this end, purified AAFs from EAEC strains C227-11 ϕcu and 042 (expressing AAF/I and AAF/II, respectively) were assessed for their ability to trigger inflammation. Both purified AAF/I and AAF/II alone failed to elicit neutrophil transmigration in our model system (Fig. S3). However, when applied to T84 cells in combination with *E. coli* K-12 strain MG1655, which alone does not induce transmigration, both types of AAF promoted PMN migration in a dose-dependent manner (Fig. 4A). The same response was observed when AAFs were combined with laboratory *E. coli* K-12 hybrid strain HB101 or the commensal *E. coli* strain F-18 (data not shown). This suggests that AAFs are sufficient to confer an inflammatory phenotype onto bacteria that are normally incapable of triggering inflammation in this model system. Neither AAF variant was found to affect adhesion of the *E. coli* MG1655 strain to T84 cells (data not shown), suggesting that the inflammatory phenotype does not result from increased binding of the bacteria.

10.1128/mBio.00717-17.3 FIG S3  Purified AAFs elicit PMN migration in the presence of commensal *E. coli* strain MG1655 but not by themselves. **, *P* < 0.01. Download FIG S3, PDF file, 0.2 MB. Copyright © 2017 Boll et al. 2017 Boll et al. This content is distributed under the terms of the Creative Commons Attribution 4.0 International license.

In keeping with results using EAEC strains, the inflammation induced by *trans*-expression of AAFs depended on MUC1 at the cell surface. As shown in Fig. 4D, MUC1 knockdown significantly reduced the ability of MG1655 to induce PMN migration in the presence of AAFs. Taken together, these findings strongly support our hypothesis that EAEC triggers inflammation through interactions between AAFs and MUC1.

Although purified AAFs alone were unable to induce PMN migration, it remains possible that AAFs alone are capable of eliciting inflammation but that they need to function in the spatial and structural context of a bacterial cell. In an attempt to mimic the structure of AAFs bound to the bacterial surface, we coated beads (Polybead polystyrene microspheres) with purified AAF/II and verified coating by immunoblot analysis (Fig. S4). However, as shown in Fig. 4B, AAF-coated beads failed to induce PMN migration. We next assessed whether AAFs require the presence of live *E. coli* to trigger PMN migration. As shown in Fig. 4C, heat-killing *E. coli* MG1655 prior to mixing with AAFs and infecting T84 cells abolished the inflammatory phenotype. This indicates that live bacteria provide a second costimulatory signal required for triggering inflammation.

10.1128/mBio.00717-17.4 FIG S4  Polybead polystyrene microspheres coated with various concentrations of purified AAF/II were separated on a gradient polyacrylamide gel and immunoblotted using AafA Rab. Samples containing only AAF/II were included as standards. Download FIG S4, PDF file, 0.4 MB. Copyright © 2017 Boll et al. 2017 Boll et al. This content is distributed under the terms of the Creative Commons Attribution 4.0 International license.

### EAEC infection enhances MUC1 expression in human intestinal xenografts in an AAF-dependent manner.

**FIG 5:**
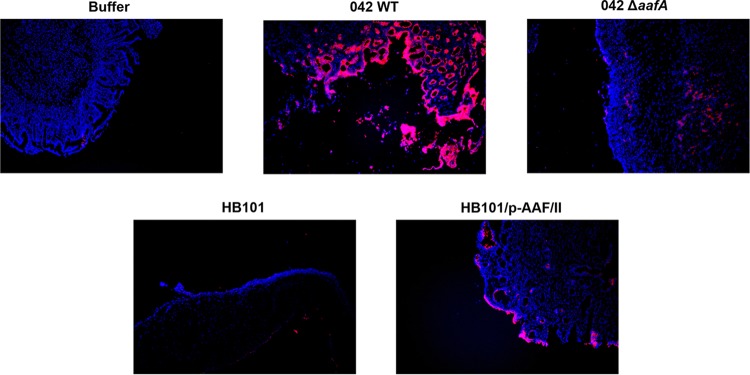
EAEC infection enhances MUC1 expression in human intestinal xenografts in an AAF-dependent manner. Human fetal small intestinal xenografts were previously infected for 24 h with wild-type 042, an 042 isogenic AAF/II mutant, *E. coli* strain HB101, HB101 carrying an AAF/II-encoding plasmid, or sterile HBSS^+^ (13). Here, tissue sections (shown in blue) were stained with DAPI, and MUC1 (shown in red) was stained using human MUC1 (VU4H5) MAb. Photographs show representative tissues for each treatment.

In contrast to colon carcinomas, MUC1 is not present at significantly high levels in healthy human colon (48). However, infection of mice with either *Campylobacter jejuni* or *Citrobacter rodentium* leads to increased MUC1 expression in inflamed intestines (26, 28). To investigate whether EAEC influences MUC1 expression in native intestinal tissues, we examined previously infected human intestinal xenografts (13) for MUC1 expression. These grafts develop into morphologically normal human intestine and secrete mucins (49). As shown in Fig. 5, staining with a human MUC1-specific antibody revealed only minimal MUC1 expression in the grafts treated with buffer or infected with the HB101 strain. In contrast, in grafts infected with the wild-type 042 strain, MUC1 expression was highly elevated at the epithelial surface. Infection with an HB101 strain expressing AAF/II-encoding genes also increased MUC1 expression, thus strongly suggesting that AAFs are sufficient to trigger elevated MUC1 expression on the intestinal epithelium.

## DISCUSSION

EAEC has gained increasing attention in recent years with a rise in outbreaks and recognition of its importance in multiple clinical settings. However, while much is known about EAEC pathogenesis, a key missing step has been the lack of identification of a host cell receptor on the surface of the intestinal epithelium. Here, we have identified the transmembrane mucin MUC1 as one such receptor.

We demonstrate that binding of EAEC to MUC1 is mediated by the AAFs, which serve multifunctional roles in the pathogenesis of this organism. Previous studies have demonstrated binding of AAFs to other cell surface components, including the extracellular matrix proteins fibronectin and laminin (21, 23). The major pilin subunits of AAF/I and AAF/II are both highly positively charged at physiologic pH, and the attachment of these two AAF variants to fibronectin is primarily based on electrostatic interactions (21). This raises the question of whether the AAF-MUC1 interactions are of a similar nature. The MUC1 glycans feature terminal sialic acids, which contribute negative charge. We found that pretreatment of MUC1-expressing HEK293 cells with neuraminidase (to release sialic acid) reduced binding of EAEC strain 042 to the cells. However, residual binding was still observed, which could indicate that sialic acid is one component of a larger oligosaccharide structure involved in EAEC binding.

Interestingly, we found that MUC1 is not the only transmembrane mucin to which EAEC binds. Adherence of EAEC strains expressing AAF variant I, II or III to T84 cells was attenuated when MUC13 expression was reduced (unpublished observations). In contrast, MUC1 reduction significantly attenuated binding of only the 042 strain (which expresses AAF/II). Binding of the C1010-00 strain, expressing AAF/IV, was not affected by reduction of either MUC1 or MUC13. It is possible that the more neutral surface charge of the AAF/IV major pilin subunit makes this particular adhesin less adept at binding mucins (17), or that redundancy prevented a single mucin knockdown from affecting C1010-00 binding. Further studies will elucidate the exact nature of the AAF-mucin interactions and the role of the oligosaccharide side chains.

In addition to potentially serving as decoys for microbes that penetrate the outer mucus layer, transmembrane mucins are involved in complex biological processes, including inflammatory signaling pathways (34). Previous work from our group has demonstrated that EAEC induces AAF-dependent migration of PMNs across intestinal epithelium (13). We have now identified MUC1 as a signaling surface receptor for AAFs and have demonstrated that EAEC exploits MUC1 to trigger PMN migration. In fact, reduced MUC1 expression attenuated the inflammatory response to EAEC strains regardless of the AAF variant expressed. While other transmembrane mucins also have signaling roles, reduced MUC13 expression did not impact EAEC-induced PMN migration in our model. This finding suggests that MUC1 plays a unique and critical role in mediating host inflammatory responses to EAEC and may act as a sensor of bacterial infection that triggers host cells to mount an inflammatory response.

Interestingly, we observed that purified AAFs combined with noninflammatory commensal *E. coli* strains could also drive PMN migration in a MUC1-dependent manner. Purified AAFs were unable to elicit inflammation by themselves, instead requiring the presence of live bacteria. This implies that bacteria provide a costimulatory signal required for the AAFs to activate cellular signaling. The fact that boiling the commensal *E. coli* prior to infection in the presence of AAFs abolished the inflammatory phenotype suggests that the costimulatory signal is heat-labile, ruling out molecules such as lipopolysaccharide (LPS) and peptidoglycan unless delivered via a mechanism that requires live bacteria. We also considered the possibility that other bacterial secreted compounds might provide a secondary signal required for AAFs to trigger the inflammatory response. However, we did not detect PMN migration following exposure of T84 cells to *E*. *coli* MG1655 supernatants in the presence or absence of AAF/II (data not shown). Moreover, we have previously shown that the 042Δ*fliC* strain, which lacks flagella, is equally capable as the 042 wt strain of inducing PMN migration, thereby ruling out flagellin out as a candidate (13). Last, we examined whether type 1 fimbriae, which bind to d-mannose residues on eukaryotic surfaces, could be involved. These adhesins are highly common in both commensal *E*. *coli*, including MG1655, and pathogenic *E. coli* and have been implicated in inflammation (50). Nevertheless, pretreatment with 1% mannose did not reduce the ability of the 042 wt or MG1655 strain in the presence of AAF/II to drive PMN migration (data not shown). However, type 1 fimbriae could still prove to be the costimulatory inflammatory signal, e.g., by mannose-independent host cell receptor recognition of the fimbriae. Further studies are ongoing in an attempt to identify the exact nature of this costimulatory signal.

MUC1 has been implicated in numerous cell signaling pathways, including those related to inflammation (32). MUC1 signaling can be mediated through phosphorylation of its cytoplasmic tail by growth factor receptors in response to external stimuli such as hypoglycosylation or bacterial contact, which in turn may lead to phosphorylation of kinases anchored to the tail of the glycoprotein. For instance, *P. aeruginosa* flagellin has been shown to bind to airway epithelial MUC1, leading to extracellular signal-regulated kinase (ERK) activation (51). External stimuli may also cause the tail to be cleaved and localized to the cell nucleus in complex with other proteins leading to transcriptional activation of proliferative and inflammatory genes, including the MUC1 gene itself (32, 52).

Most studies linking MUC1 to inflammation suggest an anti-inflammatory function. For instance, MUC1 inhibits nuclear factor NF-κB activity and interleukin-8 (IL-8) secretion in gastric epithelium in response to *H. pylori* infection (53). MUC1 also downregulates neutrophil influx in airway epithelium following *P. aeruginosa* infection (45). In another study, MUC1 suppressed conjunctivitis in mice caused by *Staphylococcus* and *Streptococcus* species (54). Our results identifying a proinflammatory role for MUC1 in response to an intestinal pathogen are novel and suggest that the role of MUC1 in inflammation is complex and likely depends on a variety of factors, including the specific pathogen and the site of infection. EAEC infection of epithelial cells triggers AAF-dependent basolateral release of IL-8, the prototypical neutrophil-attracting chemokine, facilitating recruitment of neutrophils out of the vasculature (12). We therefore sought to determine whether MUC1 also plays a role in mediating this event. However, no significant difference in IL-8 secretion was observed following EAEC 042 infection of MUC1-reduced T84 cells compared to control cells (unpublished observations). Thus, it appears that a distinct EAEC-activated host cell pathway is required for recruitment of neutrophils to the basal membrane.

We have not yet determined how AAF binding to MUC1 drives intestinal inflammation, but our understanding of how EAEC infection induces neutrophil influx suggests possible hypotheses. EAEC triggers epithelial synthesis of the PMN chemoattractant hepoxilin A_3_ (HXA_3_) in a conserved manner also utilized by *Salmonella enterica* serovar Typhimurium (41). In the case of S. Typhimurium, HXA_3_ is secreted through the apically located efflux transporter MRP2, and localization of MRP2 to the apical surface is mediated by the ERM (ezrin, radixin, and moesin) protein ezrin (55). Notably, ezrin has been shown to colocalize with MUC1 in breast carcinomas (56). The MUC1 cytoplasmic tail has also been shown to associate with protein kinase C-δ (PKC-δ), which we have previously implicated in EAEC-induced PMN migration (41, 57). Further studies will determine whether AAF binding to MUC1 is associated with changes in ezrin and PKC-δ localization or phosphorylation in a way that drives HXA_3_/MRP2 inflammation during EAEC infection.

While MUC1 is found on all epithelial surfaces, it is sparse in the healthy human intestine (48). However, MUC1 has been found to be upregulated in the inflamed intestines of mice infected with enteric pathogens as well as in human patients with ulcerative colitis (26, 28, 58). We found that infection with EAEC 042 strongly upregulated epithelial MUC1 expression and that stimulation of MUC1 expression depended on bacterial expression of AAFs. Thus, it is possible that EAEC is also capable of inducing enhanced MUC1 expression in infected individuals and that the pathogen can exploit this to its own advantage by inducing a proinflammatory neutrophil response that promotes bacterial pathogenesis. In conclusion, we have identified MUC1 as a cellular receptor for EAEC and demonstrated for the first time that MUC1 mediates a proinflammatory effect in response to an intestinal pathogen.

## MATERIALS AND METHODS

### Bacterial strains and growth conditions.

The bacterial strains used in this study are listed in Table 1. Bacteria were cultured in Luria-Bertani (LB) broth or agar. For immunofluorescent staining, EAEC prototype strains 042, 042Δ*aafA*, C1010-00, 17-2, and 55989 were transformed with pEGFP (EGFP stands for enhanced GFP) plasmid (Clontech), and carbenicillin-resistant clones were selected on LB medium.

**TABLE 1:** Bacterial strains used in this study

Strain	Description	Reference
042	Wild-type EAEC strain expressing AAF/II	59
17-2	Wild-type EAEC strain expressing AAF/I	60
JM221	Wild-type EAEC strain expressing AAF/I	19
55989	Wild-type EAEC strain expressing AAF/III	18
C1010-00	Wild-type EAEC strain expressing AAF/IV	17
042 *aafA*	Strain 042 with Tn*phoA* inserted into the *aafA* gene; previously designated mutant 3.4.14	42
C227-11 ϕcu	EAEC strain C227-11 expressing AAF/I cured of the Stx2a-bearing phage	43
IH11128	Diffusely adherent *E. coli* strain	61
MG1655	Commensal *E. coli* strain	62
F-18	Commensal *E. coli* strain	63
HB101	Nonfimbriated *E. coli* strain	64

### Cell culturing.

HEK293 cells stably expressing human MUC1 in the pcDNA3 vector (293MUC1) or empty vector pcDNA3-HEK293 (293 cells) have been previously described (39). Cells were routinely cultured in Dulbecco modified Eagle medium (DMEM), 10% heat-inactivated qualified fetal bovine serum (FBS), antibiotics, and 200 μg/ml of G418 (Sigma).

T84 cells were cultured in DMEM with nutrient mixture F-12 (DMEM–F-12) (Invitrogen) containing 10% FBS, 100 U/ml penicillin, and 100 μg/ml streptomycin. Inverted polarized T84 cell monolayers were constructed as previously described (13).

### Lentiviral transduction of T84 cells with shRNA constructs against MUC1 or MUC13.

Sequence-verified short hairpin RNA (shRNA) lentiviral plasmids in pLKO.1-puromycin vector for *MUC1* or *MUC13* silencing in human cells were obtained from Open Biosystems through the RNAi (RNA interference) Core Facility at University of Massachusetts Medical School. For production of lentiviral particles, three components were transfected into HEK 293T cells: (i) pLKO.1 vector containing shRNA; (ii) a packaging vector psPAX2 containing *gag*, *pol*, and *rev* genes; and (iii) envelope vector pMD.2G. For transfection, a mixture of 12 µg plasmid with a gene coding for shRNA plus 9 µg packaging plasmid plus 3 µg envelope plasmid combined with TransIT-293 transfection reagent (Mirus) in 1 ml Opti-MEM serum-free medium was added to HEK 293T cells plated to 80% confluence in 10-cm petri dishes. Production of lentiviruses was enhanced by replacement of the cell culture medium 24 h posttranfection. Twenty-four hours later, the viral supernatants were harvested, passed through Whatman Puradisc 0.45-µm filters, and added with 8 µg/ml Polybrene to T84 cells plated to 20% confluence in a six-well plate. After 24 h, the transduction procedure was repeated. The next day, the T84 cells were passaged into T-25 25-cm^2^ flasks containing regular T84 cell medium. Twenty-four hours later, selection for stable shRNA-containing cell was initiated by replacing medium with fresh medium containing 5 µg/ml puromycin.

### Binding assays.

Adhesion of EAEC strains to HEK293 cells was performed by two procedures. In the first procedure, HEK293 and HEK293MUC1 cells were grown on eight-well Millicell EZ glass slides (Millipore) coated with poly-l-lysine (Sigma) and incubated at 37°C for 1 h with EAEC 042 previously stained with the lipophilic probe FM4-64FX (Life Technologies, Inc.), which fluoresces upon binding to plasma membranes (red fluorescence). Subsequently, cells were washed two times with Dulbecco’s phosphate-buffered saline (PBS) (pH 7.4) and fixed with formalin for analysis by fluorescence microscopy. In the second procedure, cells were grown on monolayers, detached and rounded with PBS containing 0.35 mM EDTA, and washed with PBS. A total of 2 × 10^6^ cells in suspension were stained with FM4-64FX for membranes and 4′,6′-diamidino-2-phenylindole (DAPI) for nucleus visualization for 5 min, followed by incubation with EAEC expressing GFP at a multiplicity of infection (MOI) of 20 in DMEM without FBS and antibiotics for 1 h at 37°C. The cells were then washed three times with HBSS and resuspended in 5% formalin for analysis.

Adhesion of *E. coli* strains to T84 cells was performed by plating and CFU determination as previously described (13).

### Immunofluorescence.

Fixed HEK293 cells were blocked with PBS containing 2% FBS, incubated with fluorescein isothiocyanate (FITC)-conjugated mouse anti-MUC1 monoclonal antibody (MAb) (BD) (diluted 1:200) for 1 h, and washed three times with PBS containing Tween 20. After the cells were washed, the coverslips were mounted using ProLog antifade DAPI (Life Technologies) and PolyMount seller (Polysciences, Inc.). The immunostaining was analyzed and photographed using epifluorescence microscopy (Olympus BX51).

### Coating of polystyrene beads with purified AAF fimbriae.

AAF fimbriae from EAEC strains were purified by the method of Izquierdo et al. (23). Forty microliters of Polybead polystyrene microspheres (Polysciences Inc.) was suspended in 360 µl of 0.1 M borate buffer (pH 8.5) (Polysciences Inc.), spun down, and resuspended in borate buffer three times. Purified AAF/II or bovine serum albumin (BSA) (negative control) was then added to the beads in borate buffer (total volume of 400 µl) and incubated overnight. The coated beads were then spun down, washed three times in Hank's balanced salt solution (HBSS), and finally resuspended in HBSS to a final volume of 400 µl.

For confirming proper coating, the final resuspensions were separated on a polyacrylamide gel and immunoblotted using AafA Rab (23).

### Histological procedures on human intestinal xenografts.

Cryosections of previously infected human intestinal xenografts mounted on glass slides (13) were fixed in 4% paraformaldehyde (in PBS), washed, and blocked in 1% FBS and 0.1% Triton X-100 (in PBS), incubated overnight in blocking buffer with MUC1 (VU4H5) MAb (Abcam, Inc.) (1:100), then washed, and incubated with Alexa Fluor 633. After the slides were washed, the slides were mounted using DAPI-containing Vectashield solution (Vector) and analyzed using a fluorescence microscope.
