# Supplementary material for: Enteroaggregative Escherichia coli Adherence Fimbriae Drive Inflammatory Cell Recruitment via Interactions with Epithelial MUC1
Source: mBio. 2017 Jun 6;8(3):e00717-17. doi: 10.1128/mBio.00717-17 (PMC5461410; doi:10.1128/mBio.00717-17)
Supplement: FIG S1 [file mbo003173338sf1.pdf]

Figure S1

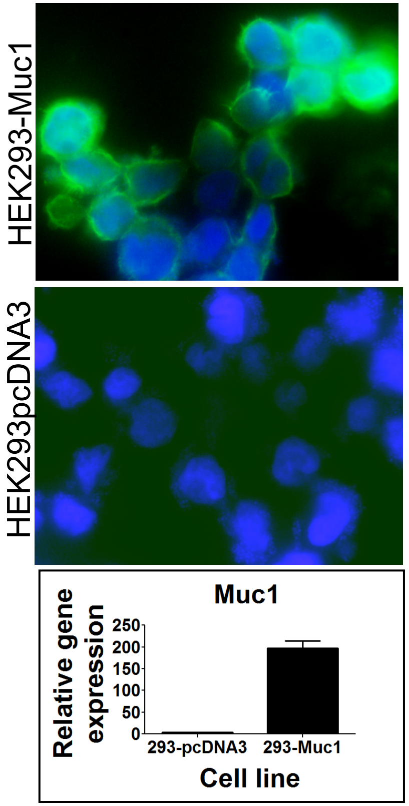

Material and methods

qRT-PCR. RNA isolation, cDNA synthesis and real-time PCR analysis of *muc1* expression in HEK293pcDNA3 and HEK293pcDNA3-MUC1 cells were performed with the following primers. MUC1F;gtgccccctagcagtaccg, MUC1R;gacgtgcccctacaagttgg, GAPDH-F; CCATGGAGAAGGCTGGGG and GAPDH-R; CAAAGTTGTCATGGATGACC as previously reported (1).

1-Hum Reprod. 2014 Aug; 29(8): 1730–1738. **Expression of the transmembrane mucins, MUC1, MUC4 and MUC16, in normal endometrium and in endometriosis.**
