# Supplementary figures and images for: Enteroaggregative Escherichia coli Adherence Fimbriae Drive Inflammatory Cell Recruitment via Interactions with Epithelial MUC1
Source: mBio. 2017 Jun 6;8(3):e00717-17. doi: 10.1128/mBio.00717-17 (PMC5461410; doi:10.1128/mBio.00717-17)

Figure S2

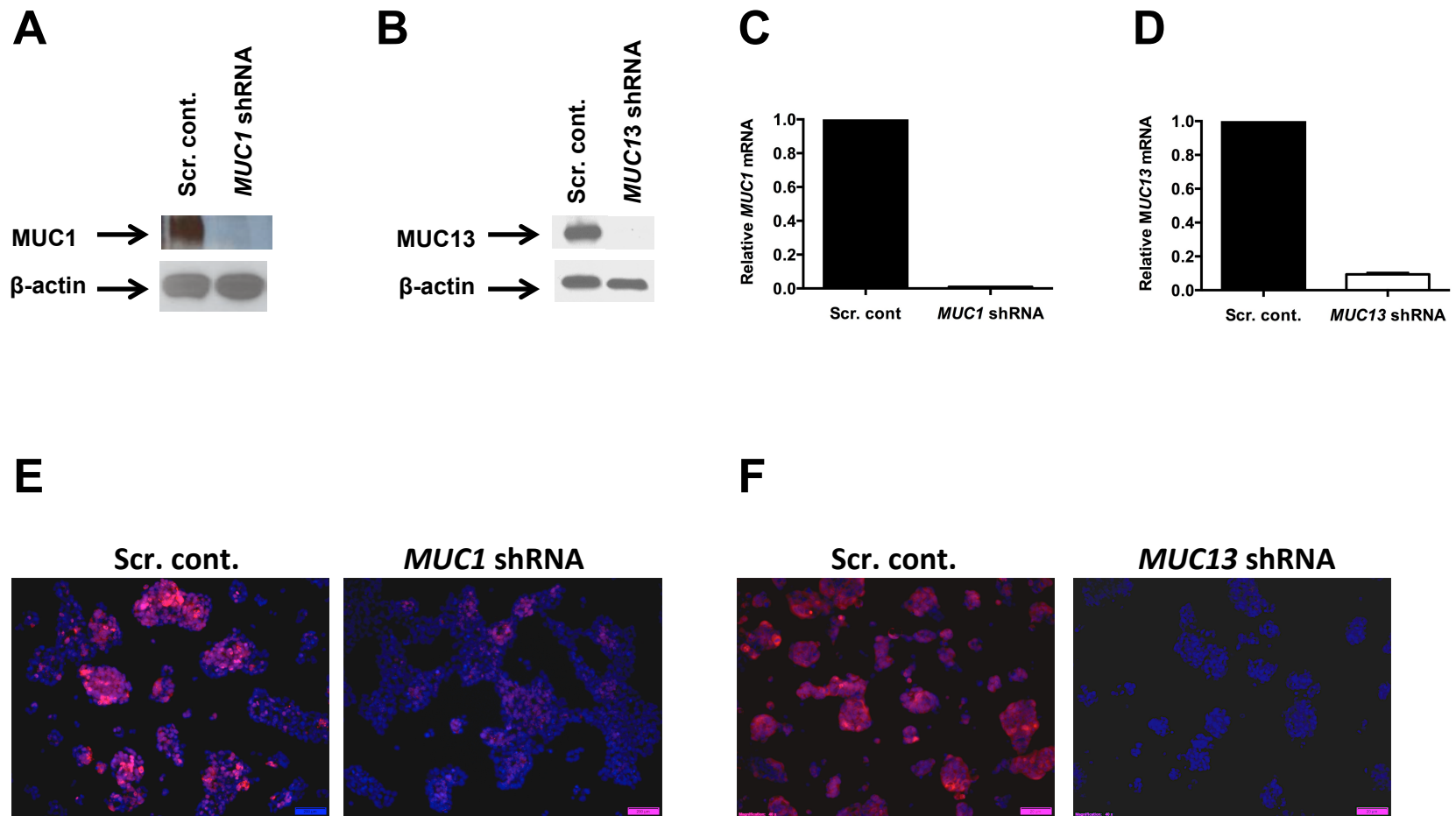

Supplement: FIG S2 [file mbo003173338sf2.pdf]

Figure S3

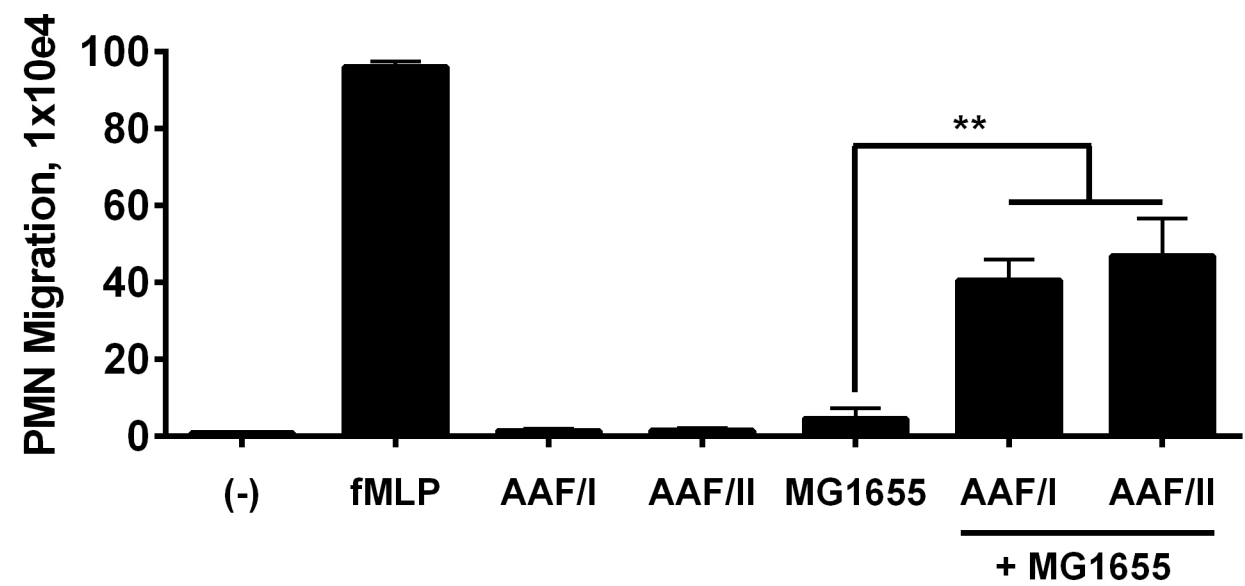

Supplement: FIG S3 [file mbo003173338sf3.pdf]

Figure S4

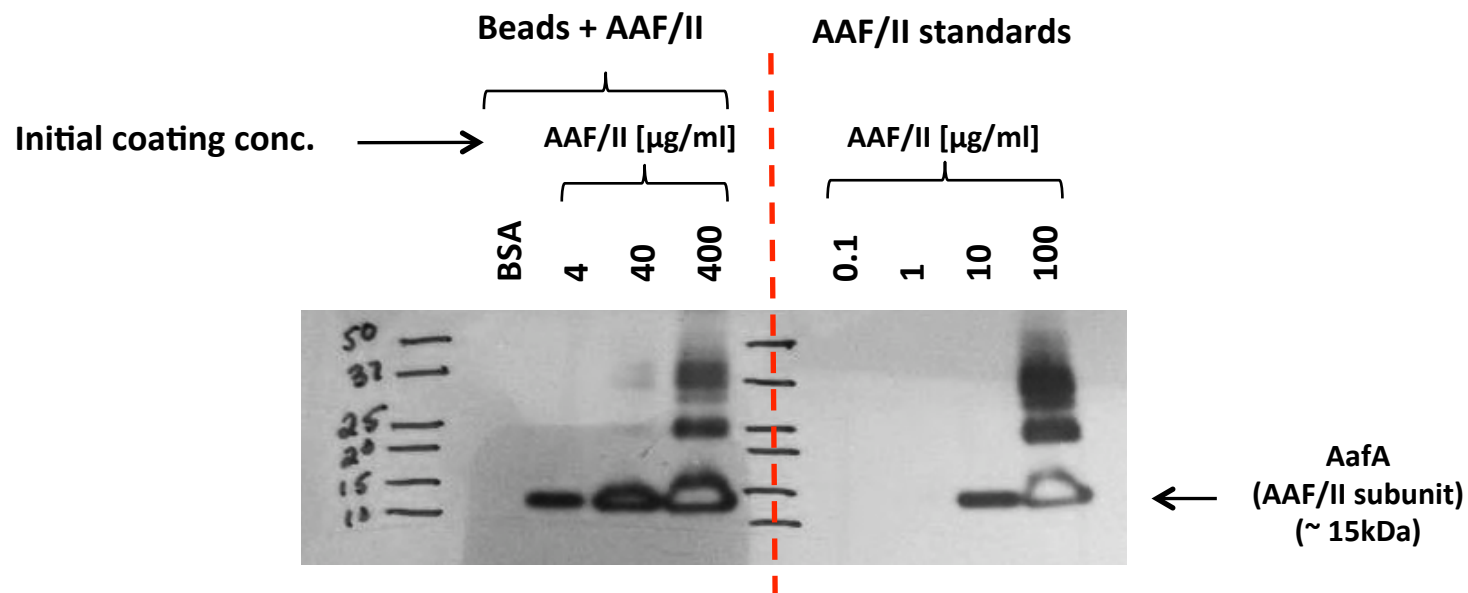

Supplement: FIG S4 [file mbo003173338sf4.pdf]
